# Supplementary material for: Periosteum-derived podoplanin-expressing stromal cells regulate nascent vascularization during epiphyseal marrow development
Source: J Biol Chem. 2022 Mar 15;298(5):101833. doi: 10.1016/j.jbc.2022.101833 (PMC9019254; doi:10.1016/j.jbc.2022.101833)
Supplement: Supporting Figures S1–S6 [file mmc1.pdf]

Periosteum-derived podoplanin-expressing stromal cells regulate nascent vascularization during epiphyseal marrow development

Shogo Tamura, Masato Mukaide, Yumi Katsuragi, Wataru Fujii, Koya Odaira, Nobuaki Suzuki, Nagaharu Tsukiji, Shuichi Okamoto, Atsuo Suzuki, Takeshi Kanematsu, Akira Katsumi, Akira Takagi, Katsuhide Ikeda, Jun Ueyama, Masaaki Hirayama, Katsue Suzuki-Inoue, Tadashi Matsushita, Tetsuhito Kojima, Fumihiko Hayakawa

List of supporting information:

1. Supporting figures (Figures S1-S6)

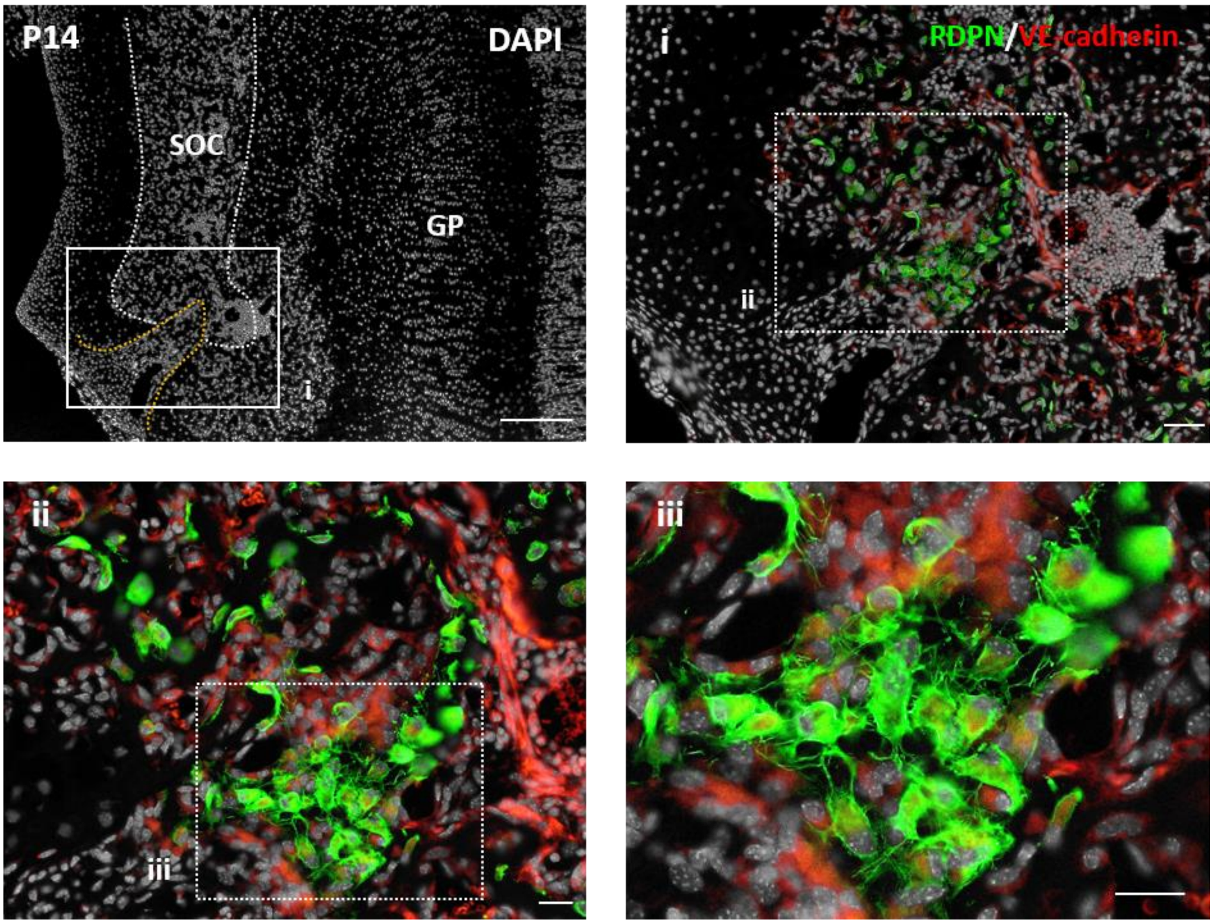

Figure S1

Proliferative expansion of podoplanin-expressing stromal cells within the penetrating of the periosteal tip. Representative IHC images of the mouse epiphysis at P14. Cryo-sections of the epiphysis were stained with PDPN, vascular endothelial (VE)-cadherin, and DAPI. Orange dotted lines indicate the periosteal penetrating tip. Scale bars in the upper left indicate 200 μm. Scale bars in the upper right, lower left, and lower right indicate 50 μm. IHC, immunohistochemistry; P14, postnatal day 14; PDPN, podoplanin; DAPI, 4′,6-diamidino-2-phenylindole; GP, growth plate; SOC, secondary ossification center

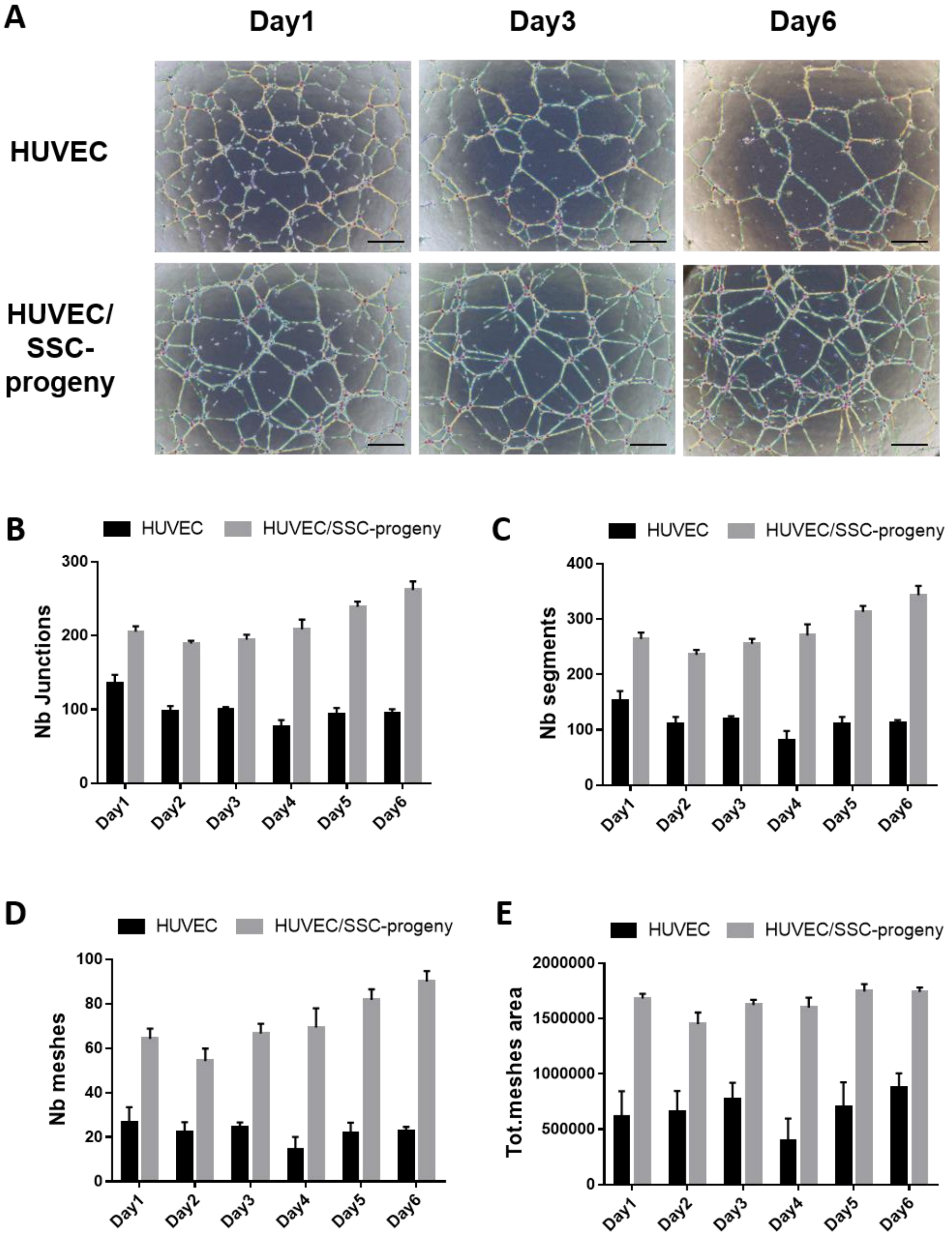

Figure S2

A long-term study of the xenovascular model co-cultured with HUVECs and PDPN-expressing SSC progenies *in vitro*. (A) Time-series images of HUVEC vascular-like lumens (upper panels) and the xenovascular model co-cultured with HUVECs and PDPN-expressing SSC progenies *in vitro* (lower panels). The day and time above each image indicate the time point from the start of the culture process. Scale bars indicate 500 µm. (B-E) Quantitative analysis of the integrity of the vascular lumen in the xenovascular model. The parameters for evaluating lumen vascularization, including the number of junctions (B), the number of segments (C), the number of meshes (D), and the total mesh area (E), were measured using an Angiogenesis Analyzer tool. The error bars represent SEMs (n = 5 per group). HUVECs, human umbilical vein endothelial cells; PDPN, podoplanin; SSC, skeletal stem cell

No.	Analyte	Alternative nomenclature	Non-CM (MPI)	SSC-progeny CM (MPI)
1	ADAMTS1	METH1	-	121.36
2	Amphiregulin	AR	-	96.95
3	Angiogenin	ANG	135.44	370.97
4	Angiopoietin-1	Ang-1	759.11	956.23
5	Angiopoietin-3	Ang-3	94.78	317.44
6	Coagulation Factor III	Tissue Factor, TF	-	255.02
7	CXCL16		-	280.26
8	Cyr61	CCN1, IGFBP-10	-	1048.28
9	DLL4		-	69.78
10	DPPIV	CD26	-	-
11	EGF		-	-
12	Endoglin	CD105	-	-
13	Endostatin/Collagen XVIII		-	135.78
14	Endothelin-1	ET-1	-	-
15	FGF acidic	FGF-1, ECGF, HBGF-1	176.19	115.19
16	FGF basic	FGF-2	244.02	-
17	KGF	FGF-7	-	100.36
18	Fractalkine	CX3CL1	-	406.51
19	GM-CSF		-	-
20	HB-EGF		-	-
21	HGF	Hepatopoietin A	-	304.56
22	IGFBP-1		-	-
23	IGFBP-2		-	5355.55
24	IGFBP-3		-	1168.82
25	IL-1α	IL-1F1	-	-
26	IL-1β	IL-1F2	-	-
27	IL-10	CSIF	-	100.95
28	IP-10	CXCL10, CRG-2	-	192.02
29	KC	CXCL1, CINC-1, GROα	-	868.75
30	Leptin	OB	-	-
31	MCP-1	CCL2/JE	-	6191.13
32	MIP-1α	CCL3	-	-
33	MMP-3 (pro and mature form)		-	7197.13
34	MMP-8 (pro form)		-	161.44
35	MMP-9 (pro and active form)		-	-
36	NOV	CCN3, IGFBP-9	-	1706.53
37	Osteopontin	OPN	159.78	3614.48
38	PD-ECGF		-	116.36
39	PDGF-AA		-	104.36
40	PDGF-AB/PDGF-BB		-	-
41	Pentraxin-3	PTX3, TSG-14	140.36	1826.23
42	Platelet Factor 4	CXCL4, PF4	-	176.19
43	PlGF-2		-	325.68
44	Prolactin	PRL	-	140.19
45	Proliferin		-	-
46	SDF-1	CXCL12	-	6855.55
47	Serpin E1	PAI-1	-	2194.94
48	Serpin F1	PEDF	-	281.44
49	Thrombospondin-2	TSP-2	-	3969.36
50	TIMP-1		-	471.51
51	TIMP-4		-	-
52	VEGF	VPF	-	1208.11
53	VEGF-B	VRF	-	160.78

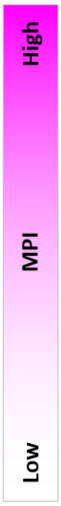

Figure S3

Quantification of spot intensities in the Proteome Profiler Mouse Angiogenesis Array Kit. Spot intensity values of the 53 angiogenic regulators present in the Non-CM medium or SSC-progeny CM medium. The MPI range is indicated using the heat distribution depicted using the color gradient. Non-CM, EGM2-basal medium supplemented with non-conditioned medium; SSC-progeny CM medium, SSC-progeny conditioned medium. SSC, skeletal stem cells; MPI, mean pixel intensity

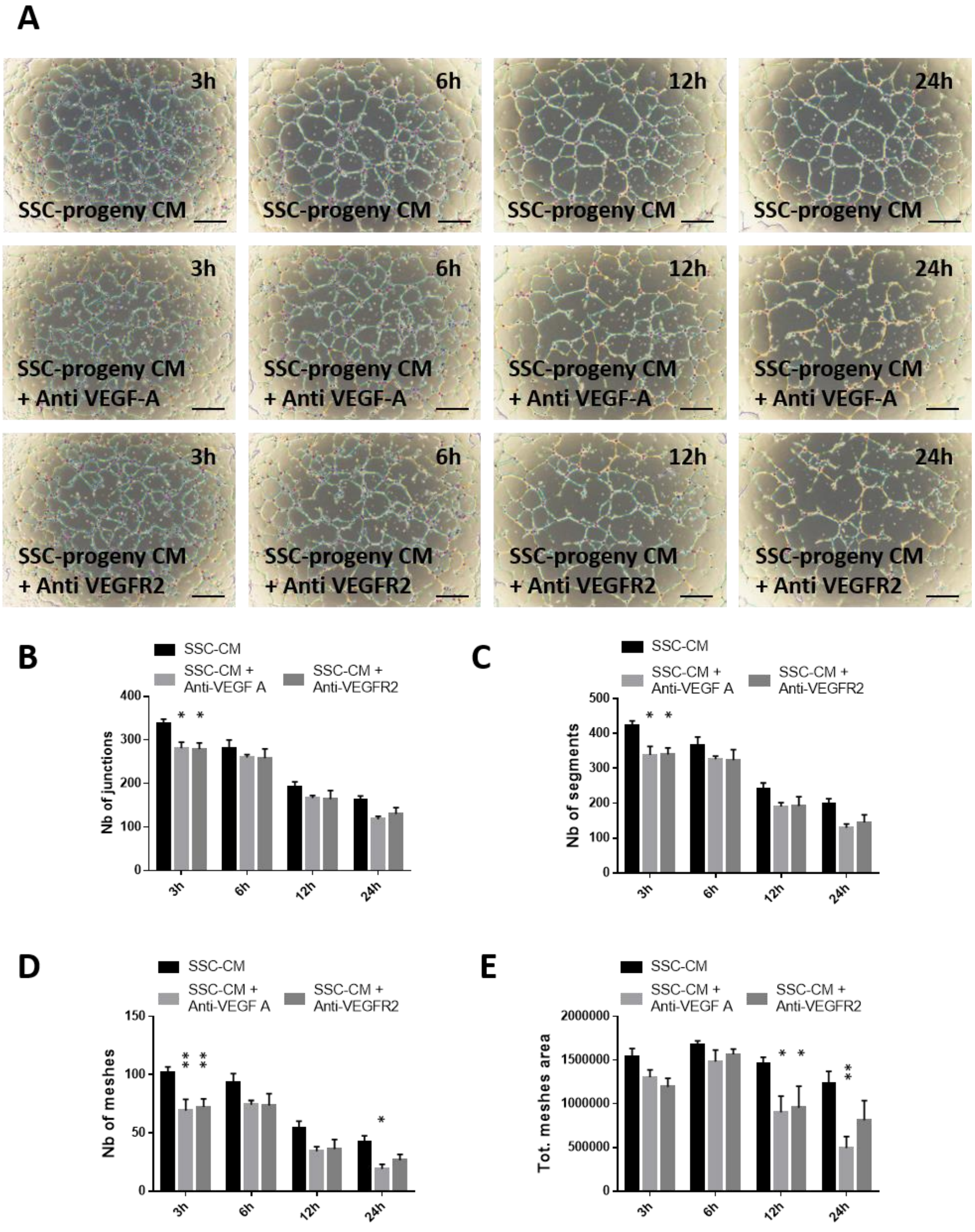

Figure S4

HUVEC tube formation assay in SSC-progeny CM with anti-VEGF-A or VEGFR2 neutralizing antibody. (A) Representative time-series images of HUVEC vascular-like lumens with SSC-progeny CM, SSC-progeny CM + anti-VEGF-A (10 mg/mL), and SSC-progeny CM + anti-VEGFR2 (10 mg/mL). Scale bars indicate 500 µm. (B-E) Quantitative analysis of HUVEC vascular lumen integrity using non-CM or SSC-progeny CM media. The parameters used for evaluating lumen vascularization, including the number of junctions (B), the number of segments (C), the number of meshes (D), and the total mesh area (E), were measured using the angiogenesis analyzer tool. *$p < 0.05$, **$p < 0.01$. Statistical analysis was performed by two-way ANOVA and Sidak's multiple comparison test (n = 5 per group). The error bars represent SEMs. HUVECs, human umbilical vein endothelial cells; SSC, skeletal stem cell

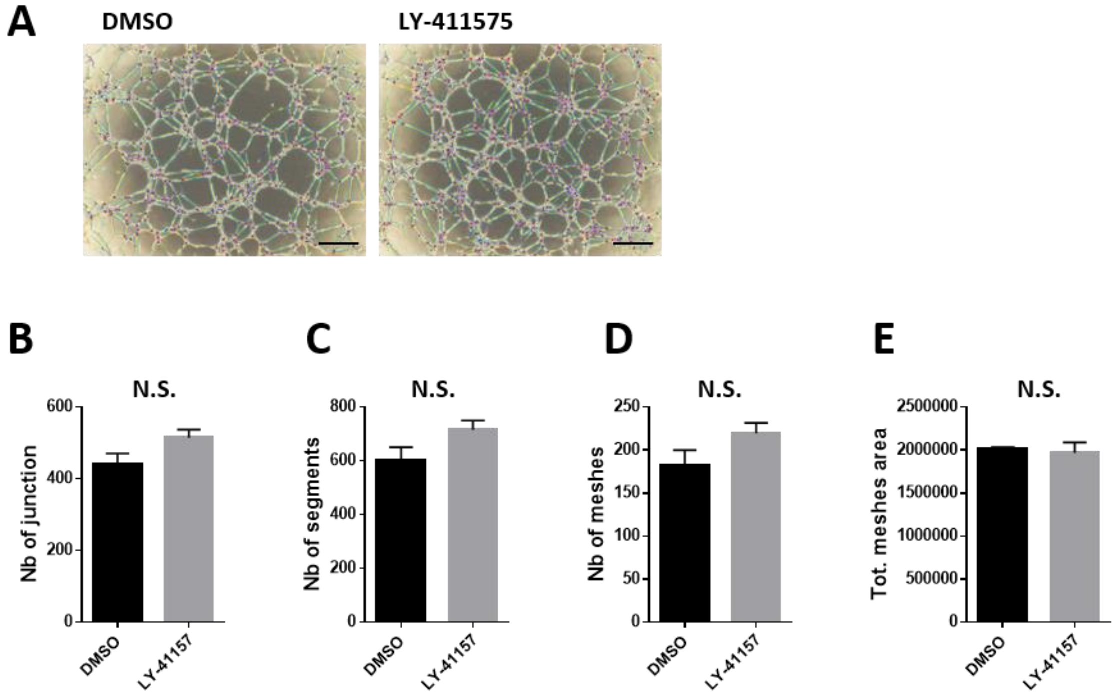

Figure S5

LY-411575 pretreatment did not affect the morphology and vascular integrity parameters of the xenovascular model. (A) Representative optical microscopic images of the xenovascular model with vehicle control (DMSO) and LY-411575. Scale bars indicate 500 µm. (B-E) Quantitative analysis of the vascular integrity. The parameters for evaluating lumen vascularization, including the number of junctions (B), the number of segments (C), the number of meshes (D), and the total mesh area (E), were measured using an Angiogenesis Analyzer tool. N.S. indicates non-significant differences. Statistical analysis was performed using the Student's t-test (n = 5 per group). The error bars represent SEMs. DMSO, dimethyl sulfoxide

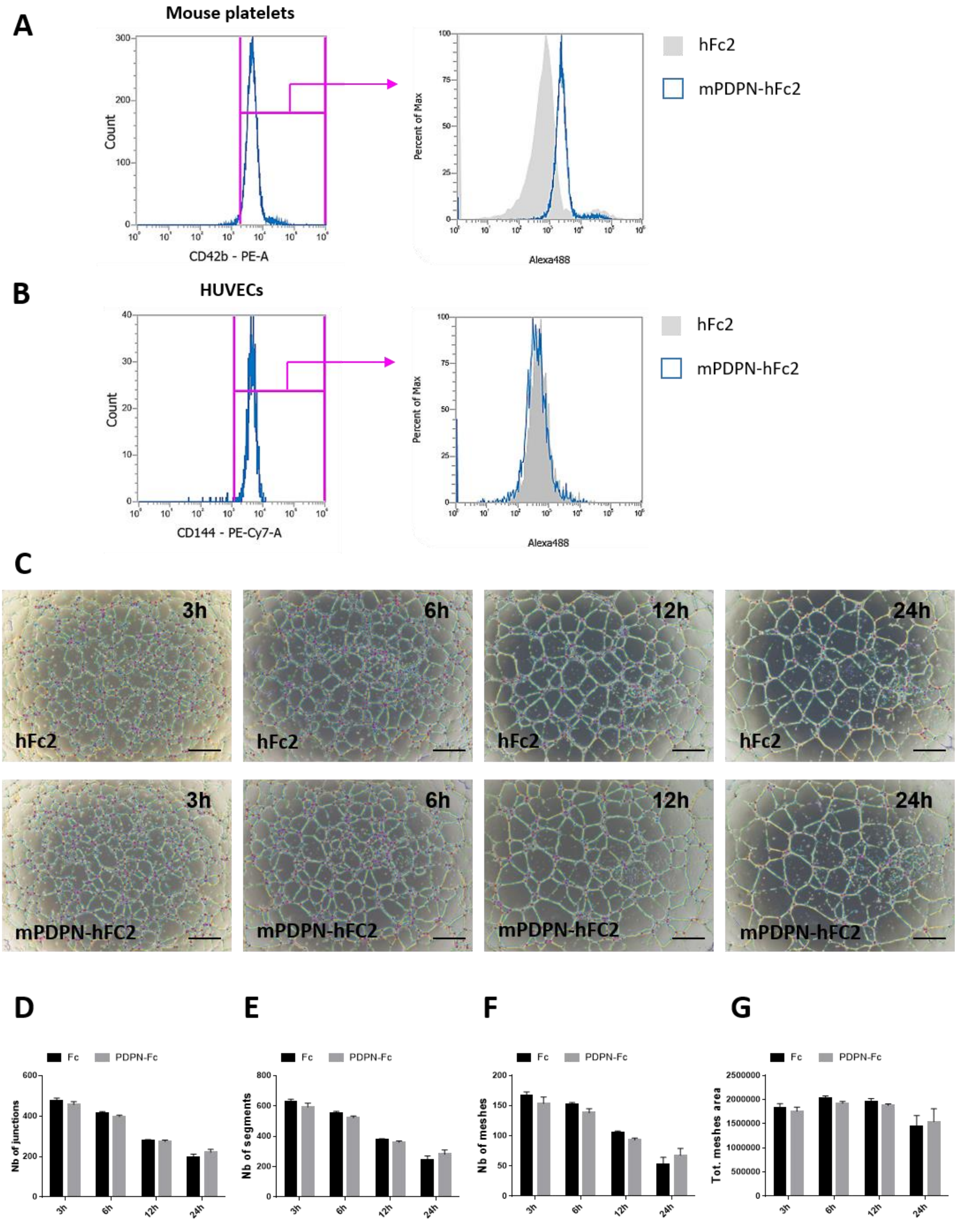

Figure. S6

Recombinant mouse PDPN-human IgG Fc2 fusion protein (mPDPN-hFc2) does not promote HUVEC vascular-like lumen integrity. (A and B) Flow cytometric analysis revealed that mPDPN-hFc2 bound to mouse platelets but not to HUVECs. Platelets express a PDPN receptor CLEC-2 and

examined as positive cells to bind mPDPN-hFc2. CD42b (GPIba) and CD144 (VE-cadherin) were used as representative markers to define platelets and HUVECs, respectively. The mPDPN-hFc2 binding to cells was detected by staining with anti-human IgG Alexa488 conjugate. (C-G) Quantitative analysis of HUVEC vascular lumen integrity with mPDPN-hFc2. (C) Representative time-series images of HUVEC vascular-like lumens with hFc2 (upper panels) and mPDPN-hFc2 (lower panels). Scale bars indicate 500 μm. (D-G) The parameters used for evaluating lumen vascularization, including the number of junctions (D), the number of segments (E), the number of meshes (F), and the total mesh area (G), were measured using the angiogenesis analyzer tool. No statistical difference was detected by two-way ANOVA and Sidak's multiple comparison test ($n = 5$ per group). The error bars represent SEMs. ANOVA, analysis of variance; HUVECs, human umbilical vein endothelial cells; PDPN, podoplanin
